# *In Vivo* Calcium Imaging of Cardiomyocytes in the Beating Mouse Heart With Multiphoton Microscopy

**DOI:** 10.3389/fphys.2018.00969

**Published:** 2018-07-31

**Authors:** Jason S. Jones, David M. Small, Nozomi Nishimura

**Affiliations:** Nancy E. and Peter C. Meinig School of Biomedical Engineering, Cornell University, Ithaca, NY, United States

**Keywords:** calcium, multiphoton microscopy, intravital, fluorescence, GCaMP

## Abstract

**Background:** Understanding the microscopic dynamics of the beating heart has been challenging due to the technical nature of imaging with micrometer resolution while the heart moves. The development of multiphoton microscopy has made *in vivo*, cell-resolved measurements of calcium dynamics and vascular function possible in motionless organs such as the brain. In heart, however, studies of *in vivo* interactions between cells and the native microenvironment are behind other organ systems. Our goal was to develop methods for intravital imaging of cardiac structural and calcium dynamics with microscopic resolution.

**Methods:** Ventilated mice expressing GCaMP6f, a genetically encoded calcium indicator, received a thoracotomy to provide optical access to the heart. Vasculature was labeled with an injection of dextran-labeled dye. The heart was partially stabilized by a titanium probe with a glass window. Images were acquired at 30 frames per second with spontaneous heartbeat and continuously running, ventilated breathing. The data were reconstructed into three-dimensional volumes showing tissue structure, vasculature, and GCaMP6f signal in cardiomyocytes as a function of both the cardiac and respiratory cycle.

**Results:** We demonstrated the capability to simultaneously measure calcium transients, vessel size, and tissue displacement in three dimensions with micrometer resolution. Reconstruction at various combinations of cardiac and respiratory phase enabled measurement of regional and single-cell cardiomyocyte calcium transients (GCaMP6f fluorescence). GCaMP6f fluorescence transients in individual, aberrantly firing cardiomyocytes were also quantified. Comparisons of calcium dynamics (rise-time and tau) at varying positions within the ventricle wall showed no significant depth dependence.

**Conclusion:** This method enables studies of coupling between contraction and excitation during physiological blood perfusion and breathing at high spatiotemporal resolution. These capabilities could lead to a new understanding of normal and disease function of cardiac cells.

## Introduction

Cardiovascular disease remains a substantial burden to populations worldwide (GBD 2013 Mortality and Causes of Death Collaborators, 2015; Mozaffarian et al., 2015). Despite significant advances in cardiovascular research, understanding of cell and tissue dynamics at a microscopic scale *in vivo* is lacking. Multiphoton microscopy (MPM) was recently demonstrated in the heart of live rodent models (Lee et al., 2012; Li et al., 2012; Jung et al., 2013; Aguirre et al., 2014; Vinegoni et al., 2015a,b). However, the combined use of intravital MPM and genetically encoded calcium indicators, which revolutionized recording of neurons (Pologruto et al., 2004; Chen et al., 2013; Prevedel et al., 2016), has not been achieved in the *in vivo*, beating heart. As a result, little is known about the interplay of *in vivo* microenvironmental features such as the microcirculation, motion, and other cellular factors, which couple and modulate function of cardiomyocytes (Rubart et al., 2003b; Wasserstrom et al., 2010; Pries and Reglin, 2017). Better understanding of the interactions between cell contraction, blood flow, and electrical conduction in disease is also needed. Previous *in vivo* studies of calcium dynamics utilized wide-field fluorescence imaging that primarily reports surface calcium transients averaged across many cells (Tallini et al., 2006; Jaimes et al., 2016). At the single-cell level, intracellular myocyte calcium and voltage transients (Rubart et al., 2003b; Wasserstrom et al., 2010; Ghouri et al., 2015), calcium dynamics of cells transplanted into myocardium (Rubart et al., 2003a, 2004; Scherschel et al., 2008; Smart et al., 2011), differences that occur with pathological hypertrophy (Tao et al., 2012), and triggered arrhythmias (Fujiwara et al., 2008) have been characterized in non-contracting Langendorff-perfusions. However, altered electromechanical function and inadequate oxygenation can drastically affect measurements of physiology in such perfused heart preparations (Kuzmiak-Glancy et al., 2015). The elimination of motion and blood flow results in highly artificial conditions and also precludes studies of the correlation between local vascular and tissue function possible in other tissues such as the brain (Shih et al., 2012; Cianchetti et al., 2013).

Here, we demonstrate MPM imaging of the genetically encoded calcium indicator GCaMP6f (Chen et al., 2013) in the beating heart within a living mouse. We show the capability to resolve calcium dynamics in single cardiomyocytes and characterize the dependence of the calcium signal on both cardiac and respiratory cycles as well as depth. Previous methods needed to compromise by limiting measurements to points in the cardiac and respiratory cycle when motion was minimized, resulting in a restricted measure of function. Our methods can measure local contraction, excitation, and vascular changes all with microscopic resolution without breath holds throughout the cardiac cycle.

## Materials and Methods

### Animals

Mice expressing the Cre-dependent GCaMP6f fast variant calcium indicator gene [B6;129S-Gt(ROSA) 26Sor^tm95.1(CAG-GCaMP6f)Hze^/J – Jackson Labs; #024105] (Chen et al., 2013) were bred on site with B6.Cg-Tg(CAG-cre/Esr1^∗^)5Amc/J (CAG-cre/Esr1) mice (Jackson Labs; #004682) (Hayashi and McMahon, 2002). Expression of GCaMP6f was induced by intraperitoneal injection of tamoxifen in corn oil (Sigma #T5648) for five consecutive days (75 mg/kg body weight), resulting in widespread tissue expression with strong expression in cardiomyocytes. C57BL/6 wild-type mice were used for control experiments. Male and female mice (25–40 g), aged 4–10 months old were used for experiments. This study was carried out in accordance with the recommendations of Guide for the Care and Use of Laboratory Animals by the National Institutes of Health. The protocol was approved by the Institutional Animal Care and Use Committee of Cornell University.

### Design of Stabilization Window and Surgery for Imaging

**FIGURE 1:**
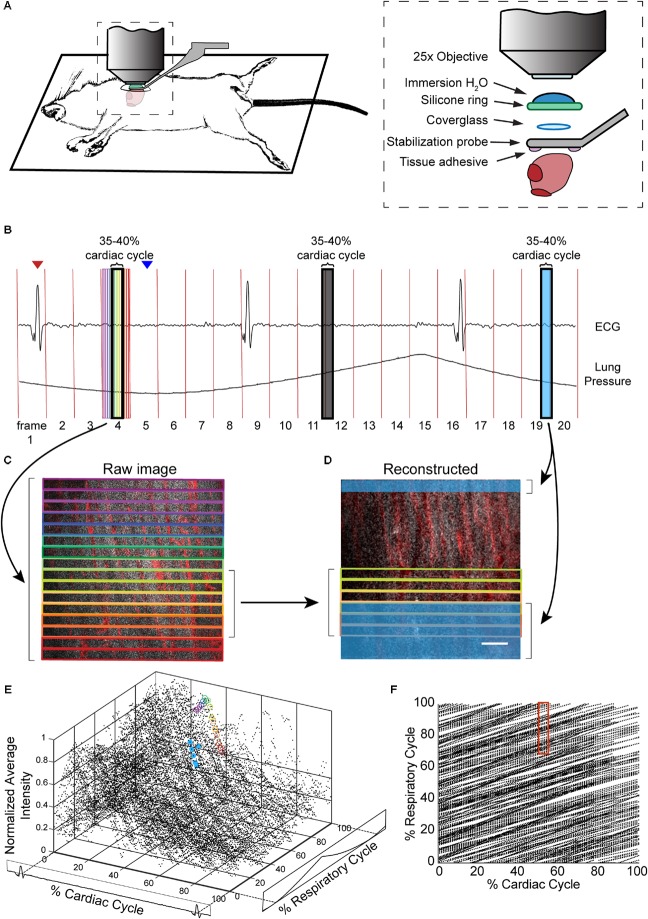
Combination of surgical approach and image reconstruction methods enable visualization and quantification of cardiac cell dynamics. **(A)** Optical access is gained via a left thoracotomy and a stabilization probe is attached to the left ventricle for microscopy. The probe holds a 3-mm diameter coverglass in contact with the heart. A silicone ring is glued to the top of the probe to hold water for the immersion of the microscope objective. Tissue adhesive is applied on the bottom surface of the probe prior to attachment to the heart. **(B)** Electrocardiogram (ECG) and ventilator pressure are recorded simultaneously during image acquisition allowing image reconstruction. Red vertical lines indicate the start of each frame; red arrow indicates the peak of R-wave used as the start of the cardiac cycle for the frame displayed below; blue arrow indicates the start of inhalation that was used as the marker of respiratory cycle. **(C)** Single raw image frames with colored boxes indicating the image segments, with corresponding timing of the acquisition indicated on the ECG and ventilator pressure traces. **(D)** A plane reconstructed using 512 × 33 pixel segments, 5% of the cardiac cycle, restricted to 70–100% of the respiratory cycle, and averaged across 4 μm in *z*. In c and d, GCaMP6f signal is shown in gray, and vasculature is shown in red. Scale bar indicates 50 μm. **(E)** GCaMP6f signal as a function of cardiac and respiratory cycles. Each data point is the intensity averaged in a 512 × 33-pixel segment with values normalized to the maximum intensity per axial slice. 50 frames acquired per *z*-plane, spanning 90 μm in *z*. Circled points correspond to data taken from regions outlined in corresponding colors in the raw image frame in **C** and in **D**. **(F)** Data density in the 2D parameter space. Each point represents a 512 × 33-pixel segment from the raw image data. The red box indicates portion of the cardiac and respiratory cycle used to generate the reconstructed plane **D**.

The design of the stabilization window and imaging setup is shown in **Figures 1A,B** and **Supplementary Figure S1**. The probe is composed of 3D-printed titanium (Materialise NV) with a 2-mm central aperture that accepts a 3-mm diameter coverslip (Deckglaser Cover Glasses, 64-0726 #0). The tissue interface side of the probe was sanded, and a channel was etched around the central aperture using fs laser ablation with 1-kHz, 15-μJ, 800-nm, 50-fs laser pulses, translated at 0.1 mm/s, and focused with a 0.28 NA microscope objective (Cerami et al., 2013). This channel prevents tissue adhesive spilling underneath the coverslip and impairing image quality. A silicone ring, fashioned from silicone molding putty (Castaldo Quick-Sil), is adhered with Loctite-406 to the top side of the window to hold water for immersion of the microscope objective at the appropriate working distance. The tail of the probe is fixed to a micromanipulator that can translate the height of the window to facilitate placement onto the ventricle wall, and provide an anchorage point for stabilization during imaging.

### Surgical Preparation

Mice were anesthetized with ketamine (10 mg/mL) and xylazine (1 mg/mL) in saline via intraperitoneal injection (0.1 mL/10 g body weight) and then intubated via the trachea using a 22-gauge, 1-inch catheter to allow mechanical ventilation (95 breath/min, 12 cm H_2_O end-inspiratory pressure; CWE Inc. SAR-830/P ventilator) with medical grade oxygen and 1.5% isoflurane for maintaining anesthesia. The ventilation rate was selected for optimum animal stability and also to avoid harmonics of the heart rate so that the cardiac and respiratory cycles were out of phase. The mouse was positioned on its right side, on top of a motorized stage with a heating pad to maintain body temperature at 37.5°C. Hair over the left thorax was depilated, skin and muscle layers over the chest wall excised, and the intercostal space between ribs 7 and 8 perforated and retracted to create space for placement of the window. Following removal of the pericardial sac, the stabilization probe was attached to the left ventricle free-wall using tissue adhesive (Vetbond). Tissue adhesive was applied to the underside of the probe and the window was gently lowered onto the left ventricle wall. A two translational-axis micromanipulator, mounted on the surgical stereotax holding the animal, provided stabilization and positioning of the probe (**Figure 1** and **Supplementary Figure S1**). Electrocardiogram (ECG) electrodes were 21 gauge needles inserted subcutaneously through the front and contralateral hind limb, connected to an isolated differential amplifier (World Precision Instruments; #ISO-80), and recorded inside a Faraday enclosure mounted to the microscope. ECG and ventilation pressure (from ventilator) signals were continuously monitored on an oscilloscope and recorded simultaneously with image acquisition. Five percent glucose in saline (0.1 mL/10 g body weight) and 0.15 mg/mL atropine sulfate (5 μg/100 g body weight) were injected subcutaneously every 30 min throughout surgery and *in vivo* microscopy. This procedure allowed stable *in vivo* imaging for 1 h. A retro-orbital injection of Texas-Red conjugated, 70 kDa dextran (50 μL, 3% in saline; Thermo #D1830) was performed to label blood plasma providing contrast in the vasculature.

### *In Vivo* Cardiac Multiphoton Microscopy

Imaging was conducted using a custom multiphoton microscope equipped with four detection channels and an 8-kHz resonant scanner (Cambridge Technology) imaged onto a galvanometric scanner pair (Cambridge Technology) to allow for both resonant and line scanning. Resonant scanning data acquisition was performed using a National Instruments digitizer (NI-5734), FPGA (PXIe-7975), and multifunction I/O module (PXIe-6366) for device control, mounted in a PXI chassis (PXIe-1073) controlled by ScanImage 2016b (Pologruto et al., 2003). A Ti:Sapphire laser (Chameleon, Coherent) with the wavelength centered at 950 nm, was used to simultaneously excite GCaMP6f and Texas-Red fluorescence. Emission was separated using a primary dichroic (Semrock FF705-Di01-49x70), a secondary dichroic (Semrock FF593-Di03-90x108), and bandpass filters selective for GCaMP6f (517/65) and Texas-Red (629/56). Water was placed within the silicone O-ring of the stabilization probe to immerse the microscope objective (Olympus XLPlan N 25 × 1.05 NA). *Z*-stack images were collected to a depth of 100–150 μm from the surface (2 μm per *z*-step) at four to five different locations per mouse. A series of 50–100 frames (1.7 to 3.3 s) per plane in *z* were collected at a scan speed of 30 frame/s.

### Assigning Cardiac and Respiratory Phase to Image Segments

The *R*-wave and expiration peaks were found using *findpeak* from the MATLAB Signal Processing Toolbox to generate a lookup table for cardiorespiratory-dependent image reconstruction and quantification. We found that with a heart rate of approximately 5 Hz and breathing at 2 Hz, ∼1.5 s or about 50 frames was sufficient to generate images in most of the cardiac/respiratory cycle space. Raw images were divided into segments of *n_block_* lines in the fast-scan axis (*x*) direction. Because the scan rate in *x*-direction (0.058 ms per line) is fast compared to the dynamics of the cardiac cell, we assigned image segments a single value of time relative to heartbeat and breathing. For all image segments, a look-up table was generated with position in *y*, position in *z*, time relative to the preceding *R* wave, time relative to the preceding trough of lung pressure, average intensity value, and a unique identifier of each heartbeat, which were used for calcium transient and image reconstruction. Phase in cardiac cycle was defined as:

$$Cardiac\;cycle\;phase=\frac{t_{seg}-R_{time_{1}}}{\max_{z}(R_{time_{2}}-R_{time_{1}})}$$

and phase in respiratory cycle was defined as:

Respiratory cycle phase=tseg−Etime1maxz(Etime2−Etime1)

where *t_seg_* is the time of a given segment, *R*_*time*_1__ is the time of the preceding *R* wave, *R*_*time*_2__ is the time of the following *R* wave, *E*_*time*_1__ is the time of the preceding minimum of pressure, *E*_*time*_2__ is the time of the following minimum of pressure, and max_z_ is the maximum occurring at a given *z* position. For exclusion of vascular regions in the quantification of fluorescence, the cardiomyocytes were identified by manually setting intensity thresholds to extracellular regions.

### Reconstruction of Image Stack

Image planes and volumes are reconstructed by sorting segments in the cardiorespiratory cycle. Each image segment within a selected range of the cardiorespiratory cycle was then registered by the position in *y* and *z* into a reconstructed stack. In cases where more than one image segment registered to the same position in the reconstruction, the redundant data was averaged. In many applications, segments with a range of *z*-positions can be averaged together if the loss in spatial resolution is acceptable. The choices of segment size, and binning in the cardiac and respiratory cycles affect the temporal and spatial resolution of the method. The binning parameters and the amount of data acquired should be varied to fit the needs of the experiment and are reported here for each set of data presented in the figures.

### Calculation of Rise Time and Tau

Intensity values from stacks were averaged over 15 μm in depth. To eliminate non-calcium dependent signals coming from autofluorescence, data from the top 15–30 μm of the raw image stack was not included so that the analysis used only frames with visible calcium transients. Due to the drop in fluorescent signal associated with inspiration, data from 25 to 50% of the respiratory cycle was excluded. Intensity was binned across 2% of the cardiac cycle and normalized to the maximum intensity in the series of images acquired at a particular depth. A single term exponential curve was fit from 90% of the peak intensity of the transient to the minimum for calculation of tau (Hammer et al., 2014). The average time from the *R* wave to the maximum value of the transient was used for the rise time.

### Image Display and Rendering

ImageJ (Schneider et al., 2012) was used to display and process reconstructed images. A median filter (1 pixel) was applied and contrast adjusted for display using only linear scaling. When displaying changes in intensity of GCaMP6f, the displayed range of values was the same at each time point. Renderings of 3D stacks were produced using Imaris x64 version 9.0.2 (Bitplane). The blend mode in Imaris was used to display the volume following the application of a smoothing filter (3 pixel × 3 pixel × 1 pixel size).

### Single-Point Motion Tracking

To quantify motion in the myocardium due to cardiac contraction, images were reconstructed by averaging over 5% of the cardiac cycle time, only using images captured during 50–100% of the respiratory cycle. To quantify motion in the myocardium due to respiratory movement, images were reconstructed averaging over 5% of the respiratory cycle, only using images captured during 50–100% of the cardiac cycle. Both cardiac and respiratory cycle reconstructions used 65 × 512 pixel segments of each frame. ImageJ was used to display reconstructed stacks combined at each 2 μm depth, with each frame of the stack representing averaged images of 5% of the cardiac or respiratory cycle. This allows the point of a small vessel bifurcation to be tracked throughout each 0–5% averaged increment of the cycle and each position (*x*, *y*, *z*) to be recorded. These measurements were repeated across three mice consisting of a total of 8 image stacks and of 14 tracked single points.

### Single-Cell Calcium Quantification

To quantify cardiomyocyte calcium transients, images were reconstructed from 512 pixel × 33 pixel segments, averaging over 2% of the cardiac cycle, using images captured during 50–100% of the respiratory cycle. Reconstructed images were averaged over 14 μm in *z*, to account for motion in *z* due to the cardiac cycle. ImageJ cardiomyocyte within the bounds of the vasculature and provide a mean intensity value at each 2% of the cardiac cycle. The Δ*F*/*F* (Δ*F*, fluorescence intensity change from fluorescence at *R* wave; *F*, fluorescence intensity at *R* wave) was calculated and filtered using the *filter* function in MATLAB with a window size of 5 and periodic boundary conditions.

### Vessel Width Quantification

To quantify changes in vessel diameter, images were reconstructed using 512 pixel × 33 pixel segments, averaging over 10% of the cardiac cycle time, using images captured during 50–100% of the respiratory cycle. *Z*-slices were maximum intensity projected in the *z*-axis to produce a single frame that was rotated so that the vessel was vertical. The line tool in ImageJ was used to take five measurements of the vessel width at 10% increments in the phase of the cardiac cycle.

### Software and Code

MATLAB was used for reconstruction and cardiorespiratory cycle-dependent analysis. Scripts and sample data are available for download at https://doi.org/10.7298/X41N7Z9D. MATLAB was used for box plots and statistical analysis. Graphpad Prism 7 was used to generate graphs of single-cell calcium traces.

### Histology

At the end of imaging, the probe was removed and the animal deeply anesthetized, followed by transcardial perfusion with cold (4°C) phosphate buffered saline (PBS, pH 7.4, Sigma-Aldrich) followed by 4% (w/v) paraformaldehyde (PFA, Thermo Fisher Scientific) in PBS. The heart was excised and cut in the cross-sectional plane at the location of the attached probe, which was indicated by remnants of tissue adhesive. Hearts were placed in 4% PFA for 1 day, and then in 30% sucrose (w/v) in PBS for 1 day. The heart was frozen in Optimal Cutting Temperature (OCT) compound (Tissue-Tek) and cryo-sectioned at 7-μm thickness onto glass slides. Hematoxylin and eosin staining was performed using standard procedures.

## Results

### Surgical Stabilization, Fast Scanning and Sorting by Cardiac and Respiratory Phase Enable *in Vivo* MPM of Cardiac Dynamics

In anesthetized, mechanically ventilated mice, we acquired ∼100-μm thick image stacks with 2-μm step size and 50–100 images per plane through a window mounted to a stabilized probe glued to the left ventricle (**Figure 1A** and **Supplementary Figure S1**) while recording the ECG and ventilator pressure (**Figure 1B**). This preparation caused minimal tissue damage (**Supplementary Figure S2a**), and heart rate was stable throughout the imaging session (**Supplementary Figure S2b**). High frame rate imaging (30 fps), using resonant scanners, produced images in real time throughout the cardiac cycle, with minimal image distortion due to tissue motion as compared to slower scanning (**Supplementary Figure S3**). In contrast to previous approaches (Aguirre et al., 2014; Vinegoni et al., 2015b), breathing was not paused during measurement and image acquisition, and the heartbeat was not synchronized to acquisition. Instead, the effects of breathing and heartbeat were decoupled by reconstructing 3D volumes from smaller image segments sorted by both the cardiac and respiratory phase (Santisakultarm et al., 2012; **Figures 1C–F**), with the size of bins in phase and position in *z* adjusted for the needs of the application.

### Volumetric Image Reconstruction Shows Both Regional and Single Cell Cardiomyocyte Calcium Transients *in Vivo*

**FIGURE 2:**
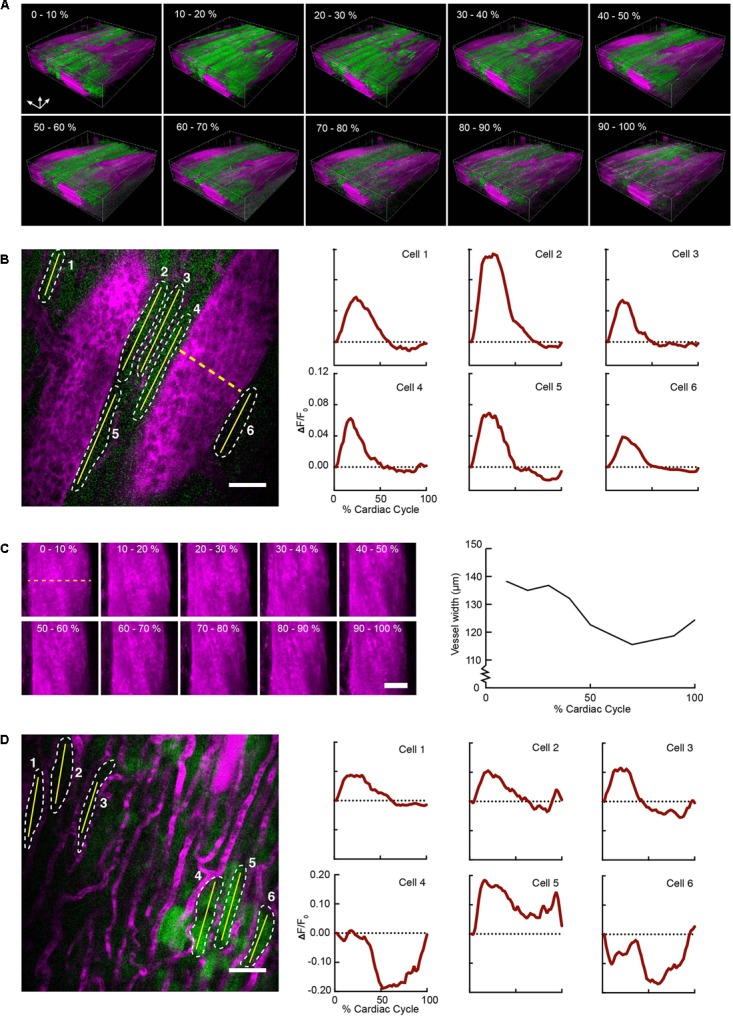
Volumetric image reconstruction enables quantification of collective and single-cell activity in the heart. **(A)** 3D renderings of image volumes reconstructed from segments of 512 × 33 pixels, at the indicated cardiac cycle phases, including 50–100% of the respiratory cycle. **(B)** GCaMP6f intensity change (Δ*F/F_0_*) in six individual cardiomyocytes (shown in a single raw image) throughout the cardiac cycle. Quantitative traces were taken from images reconstructed with 512 × 33 pixels segments, 2% of the cardiac cycle, including 50–100% of the respiratory cycle, and averaged over 14 μm in depth. **(C)** Change in vessel width over the cardiac cycle from the same imaged volume (indicated by dashed yellow line in **B**). Images show *z*-axis maximum intensity projections of images reconstructed from 512 ×33 pixel segments, averaged over 10% of cardiac cycle, including 50–100% of the respiratory cycle. **(D)** Single raw image and fluorescence changes from cells in a region near laser-induced sterile injury. Images were reconstructed as in **B** for quantitative analysis. GCaMP6f channel is shown in green, and Texas-Red dextran shows vasculature in magenta. All scale bars indicate 50 μm.

Reconstructed 3D stacks from a mouse expressing GCaMP6f in cardiomyocytes and with a vascular injection of dye enabled the clear visualization of blood vessels and cardiomyocytes and revealed the dynamic relationship throughout each phase of the cardiac cycle (**Figure 2A**). Red blood cells were visible as dark patches within the fluorescent blood plasma in the vessel lumen (Kleinfeld et al., 1998). GCaMP6f intensity changes peaked at 10–20% of the cardiac cycle. Reconstructed single frame images from the same volume enabled the GCaMP6f signal in individual cardiomyocytes to be extracted, providing quantification of calcium transients from single cells throughout the heartbeat (**Figure 2B**). As an example of a vascular measurement from the same stack, we measured changes in the width of a nearby vessel as a function of the cardiac cycle, and found width decreased to 83% (22.7 μm decrease) of its maximum diameter at ∼75% of the cardiac cycle (**Figure 2C**). Irradiation with high laser power can induce a sterile lesion that alters calcium handling (Davalos et al., 2005). In a different region in the same heart, higher power laser irradiation resulted in increased GCaMP6f fluorescence intensity compared to surrounding cells. Below the lesion, a subset of cardiomyocytes (cells 4–6 in **Figure 2D**) displayed aberrant calcium dynamics that were not synchronized with the surrounding region (cells 1–3 in **Figure 2D**).

### Cardiac- And Respiratory-Cycle Decoupling Enables Quantification of Depth-Dependent Calcium Dynamics

**FIGURE 3:**
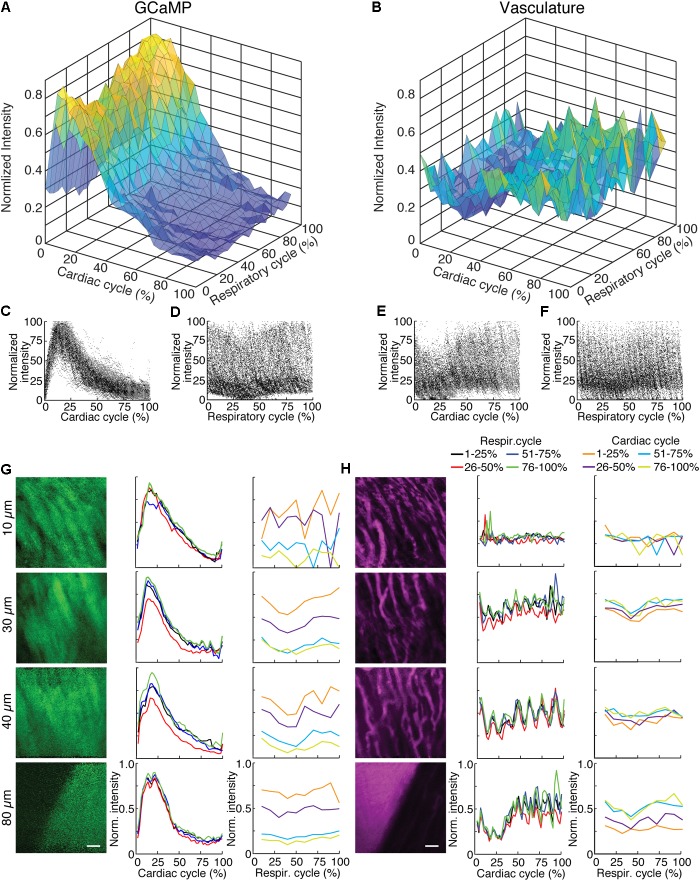
GCaMP6f imaging in ventricle wall. **(A)** Intensity of the calcium indicator GCaMP6f and **(B)** Texas-Red fluorescence channels as a function of cardiac and respiratory cycle averaged over a stack spanning 90 μm in depth. Displayed values are the average intensities from 512 × 33 pixel segments, normalized to the maximum intensity in the sequential series of raw images acquired at that plane. Data were combined in bins of 25% of both cardiac and respiratory cycle. Plots of the same data showing all individual measurements of GCaMP6f intensity across **(C)** cardiac and **(D)** respiratory cycles and from the Texas-Red channel across **(E)** cardiac and **(F)** respiratory cycles. **(G)** GCaMP6f channel and **(H)** Texas-Red channel reconstructed image projections (left), average plots of fluorescence intensities as function of cardiac phase (middle) and respiratory phase (right). Images include 50–100% of respiratory cycle, and bin 10% of the cardiac cycle, and include 10 μm in *z*. The cardiac dependence (middle) used bins of 2% of the cardiac cycle. The respiratory dependence (right) used bins of 10% of the respiratory cycle. Color of lines indicate phase range of trace in opposing cycle. Scale bars are 50 μm.

**FIGURE 4:**
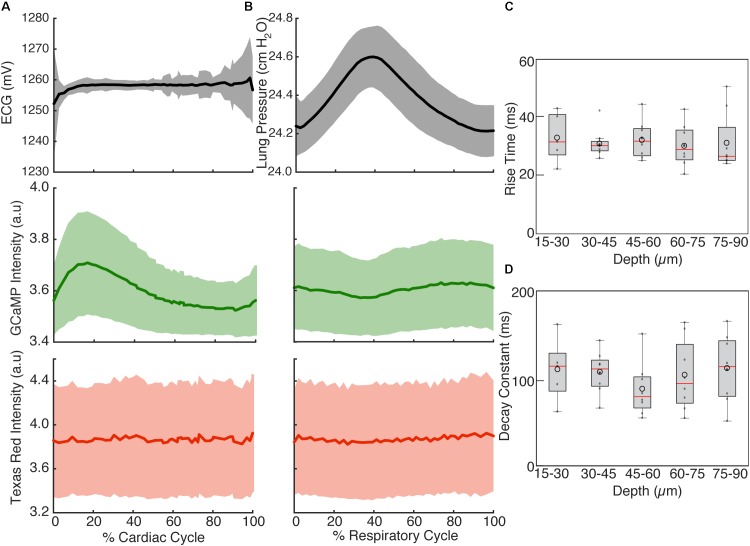
Cardiac and respiratory cycle variation in fluorescence. **(A)** Electrocardiogram voltage (ECG), fluorescence from GCaMP6f and Texas Red injected in vasculature as a function of phase in cardiac cycle. All phases of respiratory cycle were included. **(B)** Ventilator pressure, and GCaMP6f and Texas Red signal as a function of phase in respiratory cycle. All phases of the cardiac cycle were included. Solid line shows mean and shading shows standard deviation from eight stacks in three animals. **(C)** Rise time and **(D)** decay constants from the calcium responses from ten regions of interest across three animals (2% bins of the cardiac cycle, excluded 25–50% of the respiratory cycle, and binned 15 μm in depth). Box edges represent 25^th^ and 75^th^ percentiles, box centerline indicates median, and whiskers are most extreme data not considered outliers. Open circles show means. ANOVA showed no significant differences in groups (*p* = 0.67 for **C**, *p* = 0.97 for **D**).

To highlight the effects of breathing and heartbeat, GCaMP6f and vascular Texas Red fluorescence, normalized by the maximum intensity at each depth, from one imaged volume is displayed as a 2D function of both the cardiac and respiratory cycle (**Figure 3**). The GCaMP6f signal exhibited a cardiac-cycle dependent peak consistent with measurements in *ex vivo* preparations (Rubart et al., 2003b) (**Figures 3A,C**), as well as a respiratory-cycle dependent decrease near the peak lung pressure (**Figures 3A,D**). In this stack, signal from the vascular label decreased shortly after the *R*-wave and exhibited a respiration-dependent decrease that mirrored that of the GCaMP6f signal (**Figures 3B,E,F**). However, averaged over multiple stacks, Texas Red fluorescence remained constant across the cardiac cycles and nearly constant across respiratory cycles, while GCaMP6f had both cardiac and, to a lesser extent, respiratory cycle variation (**Figures 4A,B**, eight stacks in three animals). In wild type mice that did not express the calcium indicator, we imaged autofluorescence using the same emission filters used for GCaMP6f and observed a small increase in intensity within the first half of cardiac cycle (**Supplementary Figure S4a**). The un-normalized change in the fluorescence intensity, however, was about 90 times smaller than in GCaMP6f-expressing mice. To study the effect of depth, we measured the GCaMP6f and vascular label signals across both the respiratory cycle and cardiac cycle as a function of position below the heart surface (**Figures 3G,H**). We calculated rise time and decay time (Hammer et al., 2014) of the GCaMP6f signal across the cardiac cycle as a function of imaging depth (eight stacks from three mice, data from 25 to 50% of the respiratory cycle excluded to avoid confounding effects of the respiratory-dependent fluorescent intensity fluctuation) and found that both rise and decay times remained relatively constant over depths from 15 to 90 μm (*p* = 0.67, *p* = 0.97, ANOVA) with mean values of 32 ± 7 and 103 ± 34 ms, respectively, and are consistent with other measurements with GCaMP6f (Hammer et al., 2014; **Figures 4C,D**).

### Displacement in the Myocardium Is Dependent on the Cardiac and Respiratory Cycles

**FIGURE 5:**
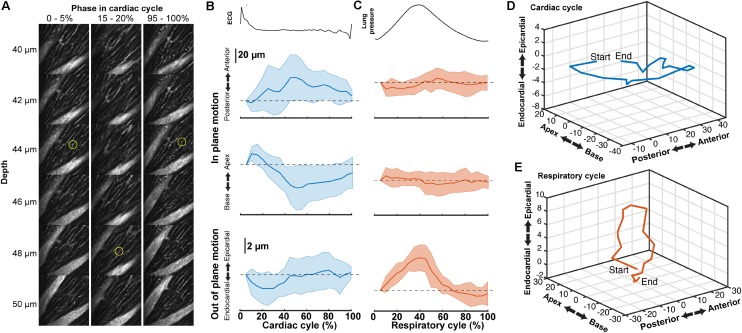
Tissue motion throughout cardiac cycle. **(A)** Representative images of myocardial vasculature labelled with Texas-Red dextran dye used for tracking the 3D motion of a structure (yellow circle) at the indicated phases in the cardiac cycle. Reconstruction used 512 × 65 pixel segments, 5% cardiac cycle bins, included 50–100% of respiratory cycle and used 2-μm bins in *z*. **(B)** Average change in the position of features in the myocardium throughout the cardiac cycle from reconstructions and **(C)** throughout the respiratory cycle (from same data, reconstructed including data 50–100% of the cardiac cycle, 5% respiratory bins). Solid lines are average and shading indicates standard deviation from 14 measurements from eight stacks in three animals. **(D)** Three-dimensional average trajectory of the same data showing the average displacement due to cardiac and **(E)** respiratory motion.

Tissue displacement relative to the stabilized probe was dependent on both heart contraction and lung inflation. To quantify this motion throughout the cardiac cycle, point-like structures, such as the edge of a capillary bifurcation, were manually tracked in 3D in reconstructed stacks (**Figure 5A**; binned by 5% of the cardiac cycle, averaged over the second half of respiratory cycle). In-plane displacements [base-apex (*x*) and anterior-posterior (*y*)] from the initial positions at the start of the cardiac cycle had an elliptical profile and were larger (32.4 ± 31.6 μm, mean ± standard deviation, and 32.3 ± 31.3 μm, respectively; 14 measurements across three animals) than out of plane in the axial (depth in myocardium) direction (3.9 ± 4.7 μm) (**Figures 5B,D**). During the respiratory cycle, displacements from the position at lowest ventilation pressure in the base-apex and anterior-posterior directions were small, while structures moved in the ventral, out-of-plane direction by 9.5 μm ± 3.8 around the time of peak lung inflation (**Figures 5C,E**; averaged over second half of cardiac cycle, 5% binning along respiratory cycle).

## Discussion

**FIGURE 6:**
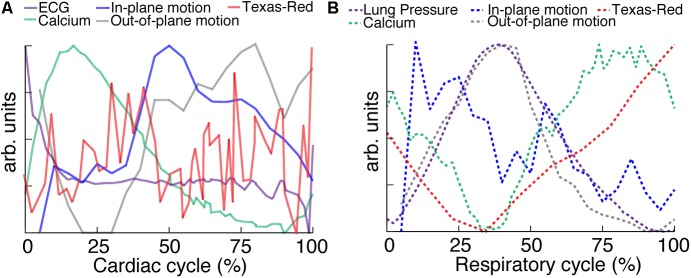
Relative timing of measured dynamics. **(A)** Summary of mean trends as a function of cardiac and **(B)** respiratory phase. In-plane motion, is calculated from means in **Figure 5** as $\sqrt{x^{2}+y^{2}}$. All curves have been normalized to the maximum and minimum.

Multiphoton microscopy of GCaMP6f-expressing mice provides a novel method to study *in vivo* excitation-contraction coupling. Using the combination of fast image acquisition, surgical stabilization, image reconstruction algorithms, and a genetically encoded calcium reporter, we demonstrated the ability to image and quantify *in vivo* calcium transients at single-cell resolution in the beating mouse heart during respiration. This enabled the measurement of aberrant calcium activity in individual cells within the tissue. Furthermore, this technique allows the simultaneous quantification of additional *in vivo* cardiac dynamics including displacement and vessel width changes at each phase of the cardiac and respiratory cycles. **Figure 6** summarizes the relative timing of physiologic dynamics over the cardiac and respiratory cycles. Data aggregated across three animals show that the GCaMP6f fluorescence peak precedes the maximum in-plane displacement but coincides with the out-of-plane motion in cardiac cycle. Such measurements enable novel assays of the relationship between excitation and mechanical output.

Multiple aspects of these measurements suggest that variation of GCaMP6f intensity across the cardiac cycle is largely attributable to changes in calcium concentration. The contribution of autofluorescence (**Supplementary Figure S4**), which is likely dominated by NADH (Zipfel et al., 2003; Blinova et al., 2004), was small compared to the GCaMP6f signal. The volume of cardiomyocytes stays approximately constant throughout contraction (Sonnenblick et al., 1967; Rodriguez et al., 1992; Leu et al., 2001), so that the concentration of GCaMP6f likely stays the same and does not contribute to the signal change. The fluorescence from the vascular label stayed constant over the cardiac cycle, although individual planes showed fluctuations both increasing and decreasing which tended to match timing of the out-of-plane motion, peaking around 25% of the cardiac cycle (for example, **Figure 3H**). The lack of cardiac-cycle dependence of the vascular channel suggests that optical or motion effects, which would be similar in both fluorescence channels, likely do not account for the change in the GCaMP6f cardiac-dependent signal. Relative to the respiratory cycle, the GCaMP6f and vascular signals had a minimum around 35% of the respiratory cycle, which also coincided with the timing of the maximum out-of-plane displacement and ventilator pressure (**Figure 6B**). While we cannot completely rule out a contribution from out-of-plane motion effects on the GCaMP6f intensity increase during the cardiac cycle, we can argue that the magnitude of the effect of cardiac cycle motion must be less than that of breathing motion because the breathing displacement is larger than the cardiac cycle displacement. This suggests that motion, at worst, can only account for a small fraction of the cardiac cycle GCaMP6f signal intensity change. Both breathing and heartbeat change the shape and composition of tissue so that future studies will need to take the resulting changes in path length, absorption, scattering, and wavefront distortion into account.

Our methods enable simultaneous measurement and visualization of multiple aspects of cardiac physiology at the cellular scale, including motion, calcium activity, and vasculature. The time resolution of the acquisition allows data collection throughout systolic contraction of the cardiomyocyte. Allowing the animal to respire during measurements enables studies of the coupling between respiratory and cardiac function and facilitates studies of disease models that could be confounded by breath holds. Although both clinical practice and experimental protocols take advantage of the coupling between breathing and cardiac function, an understanding of the relationship between the respiration and cardiac function, such as the diving reflex in which a breath hold decreases heart rate (Panneton, 2013), is still incomplete. Breathing also exerts forces on the cardiac tissue, which could influence tissue perfusion and cardiomyocyte function in both disease and healthy individuals.

In this method, there is a tradeoff between (1) the degree of binning in cardiac and respiratory phase and in the extent of spatial averaging and (2) the amount of time spent imaging to acquire sufficient data at a given depth. We demonstrated that 1.5–3 s of image acquisition per axial plane is sufficient for characterization across both respiratory and cardiac cycles, so experiments involving stacks before and after a manipulation or time lapse studies are quite feasible. Methodological limitations include the direct adherence of the window to the cardiac tissue, which, although the region of adhesive is limited as much as possible with a thin ring, could alter tissue function. This preparation does allow recovery procedures in which the probe is removed and the animal recovers, but repeated open chest surgeries are a practical limitation.

While faster imaging modalities such as ultrasound (Cikes et al., 2010) and OCT (Srinivasan et al., 2010) can measure tissue motion and blood flow more conveniently that MPM, fluorescence techniques have the advantage of the availability of many functional indicators such as GCaMP6f and label specificity through promoter-driven reporter gene expression. Combined with our protocol for *in vivo* imaging, this makes MPM a potent tool for cardiac studies.

## Author Contributions

JJ and DS designed and executed the experiments, conducted data analysis, and wrote the manuscript. NN designed the experiments and wrote the manuscript.

## Conflict of Interest Statement

The authors declare that the research was conducted in the absence of any commercial or financial relationships that could be construed as a potential conflict of interest.

## Supplementary Material

FIGURE S1 Stabilization probe. **(a)** top and **(b)** bottom views of the 3D-printed titanium stabilization probe. A femtosecond laser ablated groove on the underside that prevents tissue adhesive from leaking underneath the coverglass. **(c)** Probe attached to heart showing attached green silicone ring that serves as reservoir for the microscope objective immersion fluid. **(d)** Left side thoracotomy of the mouse presents the left ventricular wall for placement of the imaging window. **(e)** Probe attached to the heart showing the left ventricle myocardium through the imaging window. **(f)** Stabilization probe attached to the micromanipulator that allows accurate placement of the probe onto the ventricle wall.

FIGURE S2 Histology of the imaged heart. **(a)** Hematoxylin and eosin staining of *in vivo*, MPM imaged heart demonstrated no observable damage to cell structure (insert). **(b)** Heart rate during imaging remains constant.

FIGURE S3 Higher frame rate imaging shows reduced in-frame motion due to heart contraction. Raw image frames showing same cardiac vessel with **(a)** standard galvonometric scanning and **(b)** resonant scanning. Green dotted lines indicate the timing of the peak of the *R*-wave from the electrocardiogram which align with image artifacts. Scale bar is 10 μm.

FIGURE S4 Intensity as a function of respiratory and cardiac cycle in a wild type mouse analyzed in the same manner as **Figure 3**. **(a)** Autofluorescence intensity measured with the same wavelength range as for GCaMP6f and **(b)** Texas-Red fluorescence as a function of cardiac and respiratory cycle over a stack spanning 90 μm in depth. Displayed values are the average intensities from 512 × 33 pixel segments, normalized to the maximum intensity in the sequential series of raw images acquired at that plane. Data were combined in bins of 25% of both cardiac and respiratory cycle. Plots of the same data showing all individual measurements of autofluorescence intensity across **(c)** cardiac and **(d)** respiratory cycles and from the Texas-Red channel across **(e)** cardiac and **(f)** respiratory cycles. **(g)** GCaMP6f channel and **(h)** Texas-Red channel reconstructed image projections (left), average plots of fluorescence intensities as function of cardiac phase (middle) and breathing phase (right). Images include 50–100% of respiratory cycle, and 10% of the cardiac cycle, and include 10 μm in *z*. The cardiac dependence (middle) used bins of 2% of the cardiac cycle. The respiratory dependence (right) used bins of 10% of the respiratory cycle. Color of lines indicate phase range of trace in opposing cycle. Scale bars are 50 μm.
