# Supplementary figures and images for: In Vivo Calcium Imaging of Cardiomyocytes in the Beating Mouse Heart With Multiphoton Microscopy
Source: Front Physiol. 2018 Jul 31;9:969. doi: 10.3389/fphys.2018.00969 (PMC6079295; doi:10.3389/fphys.2018.00969)

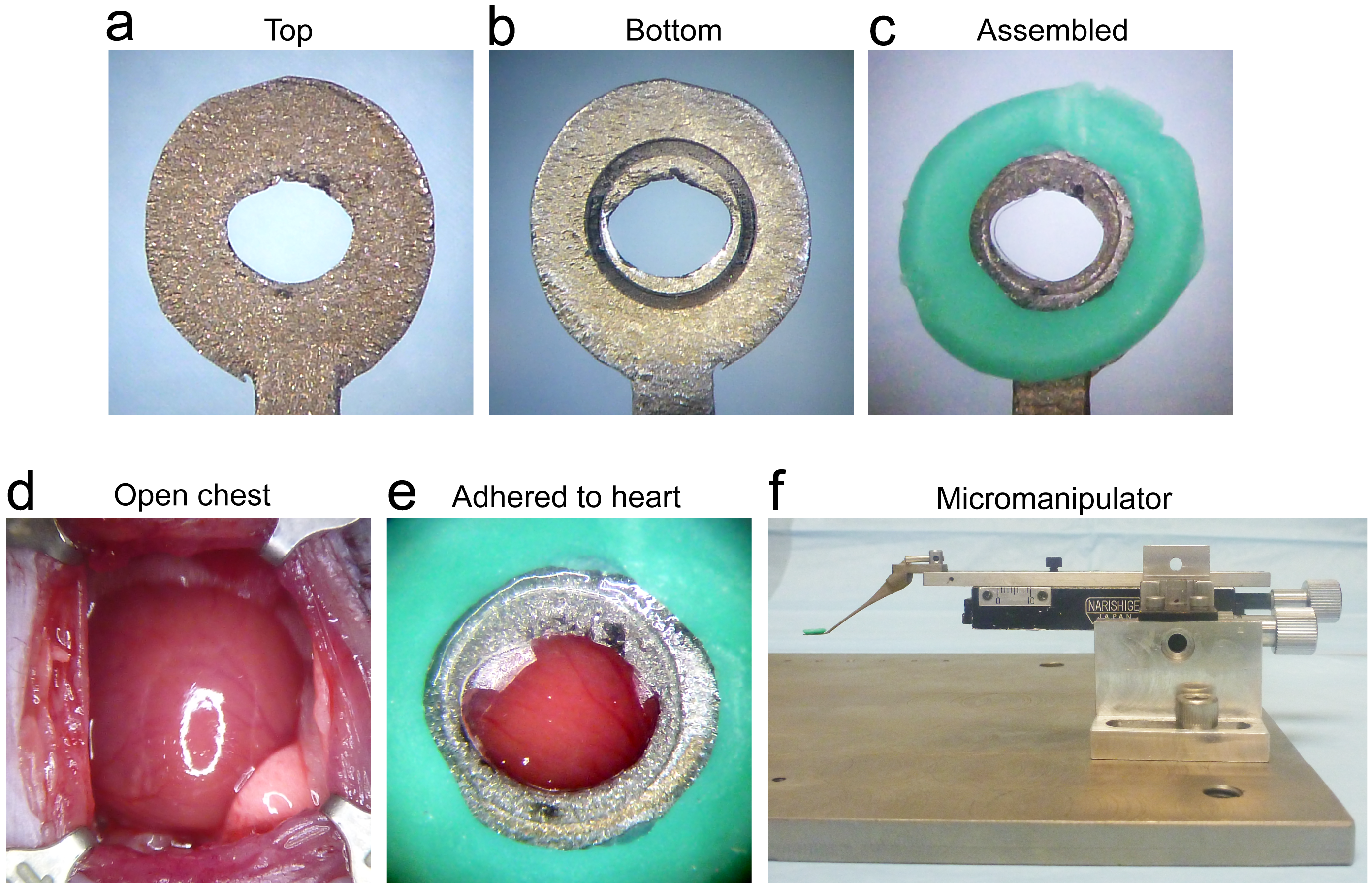

Supplement: FIGURE S1 — Stabilization probe. (a) top and (b) bottom views of the 3D-printed titanium stabilization probe. A femtosecond laser ablated groove on the underside that prevents tissue adhesive from leaking underneath the coverglass. (c) Probe attached to heart showing attached green silicone ring that serves as reservoir for the microscope objective immersion fluid. (d) Left side thoracotomy of the mouse presents the left ventricular wall for placement of the imaging window. (e) Probe attached to the heart showing the left ventricle myocardium through the imaging window. (f) Stabilization probe attached to the micromanipulator that allows accurate placement of the probe onto the ventricle wall. [file Image_1.TIF]

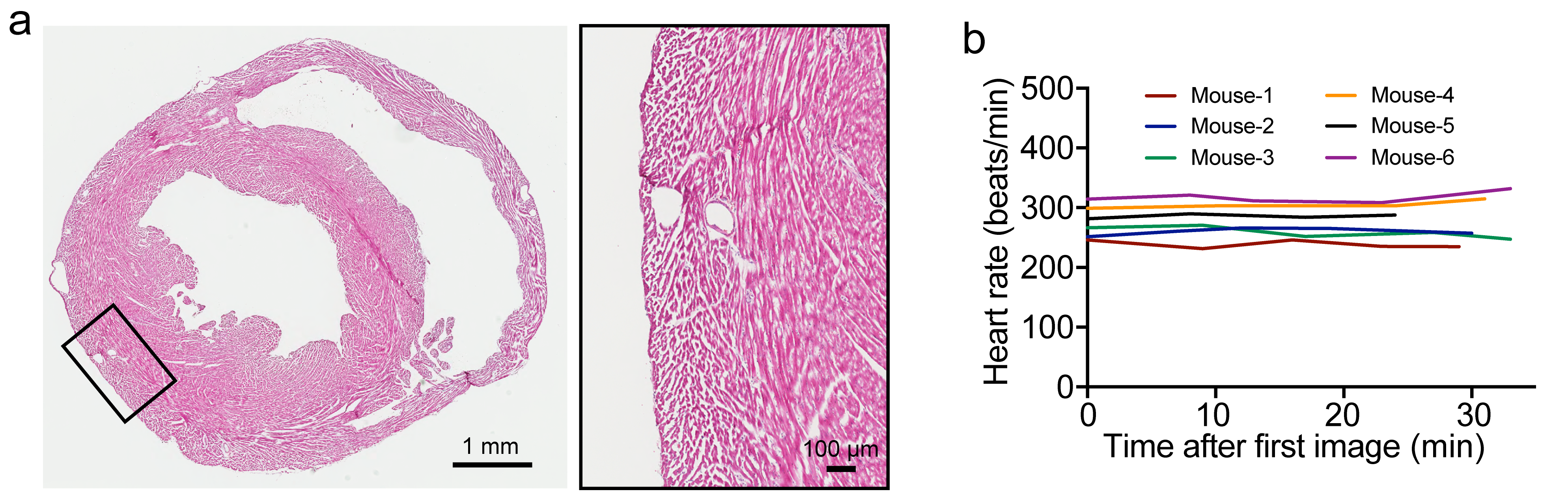

Supplement: FIGURE S2 — Histology of the imaged heart. (a) Hematoxylin and eosin staining of in vivo, MPM imaged heart demonstrated no observable damage to cell structure (insert). (b) Heart rate during imaging remains constant. [file Image_2.TIF]

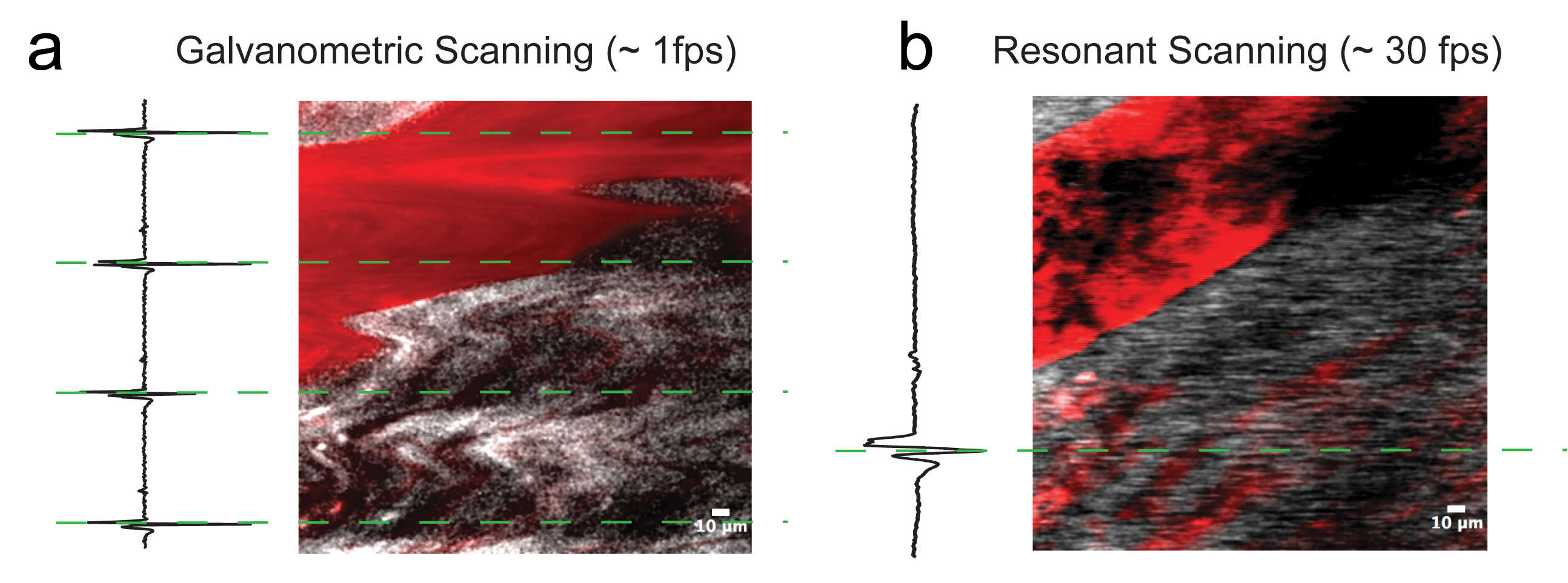

Supplement: FIGURE S3 — Higher frame rate imaging shows reduced in-frame motion due to heart contraction. Raw image frames showing same cardiac vessel with (a) standard galvonometric scanning and (b) resonant scanning. Green dotted lines indicate the timing of the peak of the R-wave from the electrocardiogram which align with image artifacts. Scale bar is 10 μm. [file Image_3.TIF]

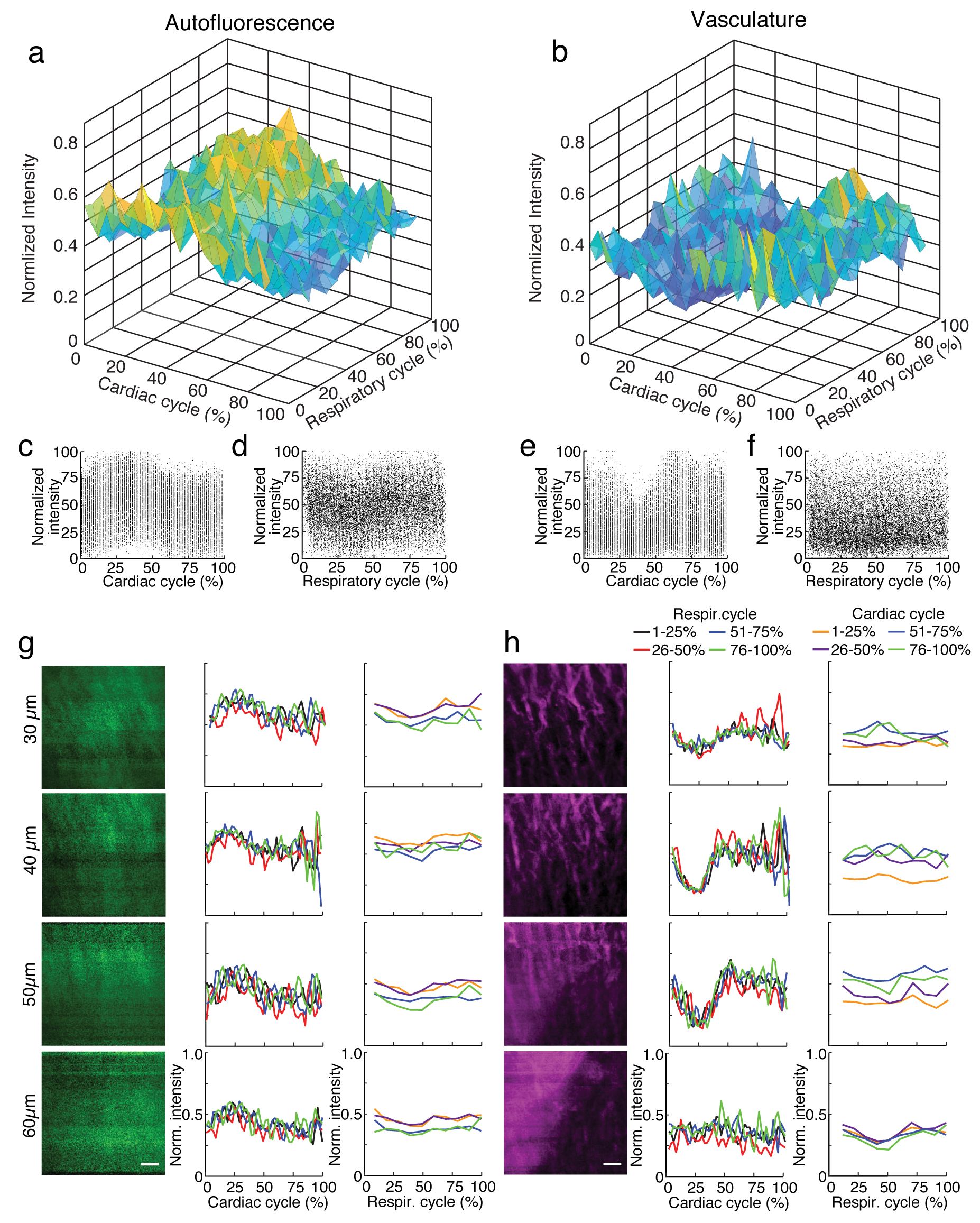

Supplement: FIGURE S4 — Intensity as a function of respiratory and cardiac cycle in a wild type mouse analyzed in the same manner as Figure 3. (a) Autofluorescence intensity measured with the same wavelength range as for GCaMP6f and (b) Texas-Red fluorescence as a function of cardiac and respiratory cycle over a stack spanning 90 μm in depth. Displayed values are the average intensities from 512 × 33 pixel segments, normalized to the maximum intensity in the sequential series of raw images acquired at that plane. Data were combined in bins of 25% of both cardiac and respiratory cycle. Plots of the same data showing all individual measurements of autofluorescence intensity across (c) cardiac and (d) respiratory cycles and from the Texas-Red channel across (e) cardiac and (f) respiratory cycles. (g) GCaMP6f channel and (h) Texas-Red channel reconstructed image projections (left), average plots of fluorescence intensities as function of cardiac phase (middle) and breathing phase (right). Images include 50–100% of respiratory cycle, and 10% of the cardiac cycle, and include 10 μm in z. The cardiac dependence (middle) used bins of 2% of the cardiac cycle. The respiratory dependence (right) used bins of 10% of the respiratory cycle. Color of lines indicate phase range of trace in opposing cycle. Scale bars are 50 μm. [file Image_4.TIF]
